# MicroRNA gene expression signatures in long-surviving malignant pleural mesothelioma patients

**DOI:** 10.1016/j.gdata.2016.06.009

**Published:** 2016-06-20

**Authors:** Ruby C.Y. Lin, Michaela B. Kirschner, Yuen Yee Cheng, Nico van Zandwijk, Glen Reid

**Affiliations:** a Asbestos Diseases Research Institute, Sydney, Australia; b School of Medical Sciences, University of New South Wales, NSW, 2052, Australia; c Division of Thoracic Surgery, University Hospital Zurich, 8091 Zurich, Switzerland; d Sydney Medical School, University of Sydney, NSW, 2000, Australia

**Keywords:** microRNA, Mesothelioma, Pathway, Systems biology, Therapeutic agents

## Abstract

Malignant pleural mesothelioma (MPM) is a tumor originating in the mesothelium, the membrane lining the thoracic cavities, and is induced by exposure to asbestos. Australia suffers one of the world's highest rates of MPM and the incidence is yet to peak. The prognosis for patients with MPM is poor and median survival following diagnosis is 4–18 months. Currently, no or few effective therapies exist for MPM. Trials of targeted agents such as antiangiogenic agents (VEGF, EGFR) or ribonuclease inhibitors (ranpirnase) largely failed to show efficacy in MPM Tsao et al. (2009) [1]. A recent study, however, showed that cisplatin/pemetrexed + bevacizumab (a recombinant humanized monoclonal antibody that inhibit VEGF) treatment has a survival benefit of 2.7 months Zalcman et al. (2016) [2]. It remains to be seen if this targeted therapy will be accepted as a new standard for MPM. Thus the unmet needs of MPM patients remain very pronounced and almost every patient will be confronted with drug resistance and recurrence of disease. We have identified unique gene signatures associated with prolonged survival in mesothelioma patients undergoing radical surgery (EPP, extrapleural pneumonectomy), as well as patients who underwent palliative surgery (pleurectomy/decortication). In addition to data published in Molecular Oncology, 2015;9:715-26 (GSE59180) Kirschner et al. (2015) , we describe here additional data using a system-based approach that support our previous observations. This data provides a resource to further explore microRNA dynamics in MPM.

**Table:** 

Specifications	
Organism/cell line/tissue	Human malignant mesothelioma tissue (micro-dissected using laser capture)
Sex	Male 75%, female 25% (2 females in long and short group respectively)
Sequencer or array type	Agilent unrestricted AMADID miRNA 8x15k-AMADID:021827 miRNA array
Data format	Log2 transformation and normalized to the 90th percentile without baseline transformation.
Experimental factors	Short-term vs long-term survival, without prior chemotherapy
Experimental features	Differential gene expression of microRNAs were selected and evaluated against clinical data of survival outcome to determine their prognostic nature.
Consent	Waiver of consent for these patient samples was granted by the Human Research Ethics Committee at Concord Repatriation General Hospital, Sydney, Australia (CH62/6/2009/078). The histopathology of all samples was independently reassessed by Assoc Prof Sonja Klebe, an expert pathologist and final diagnoses were made according to World Health Organization criteria [4].
Sample source location	Sydney, Australia

## 1 Direct link to deposited data

MicroRNA profiling of malignant pleural mesothelioma tumour tissues, http://www.ncbi.nlm.nih.gov/geo/query/acc.cgi?acc=GSE59180

## 2 Experimental design, materials and methods

**Fig. 1:**
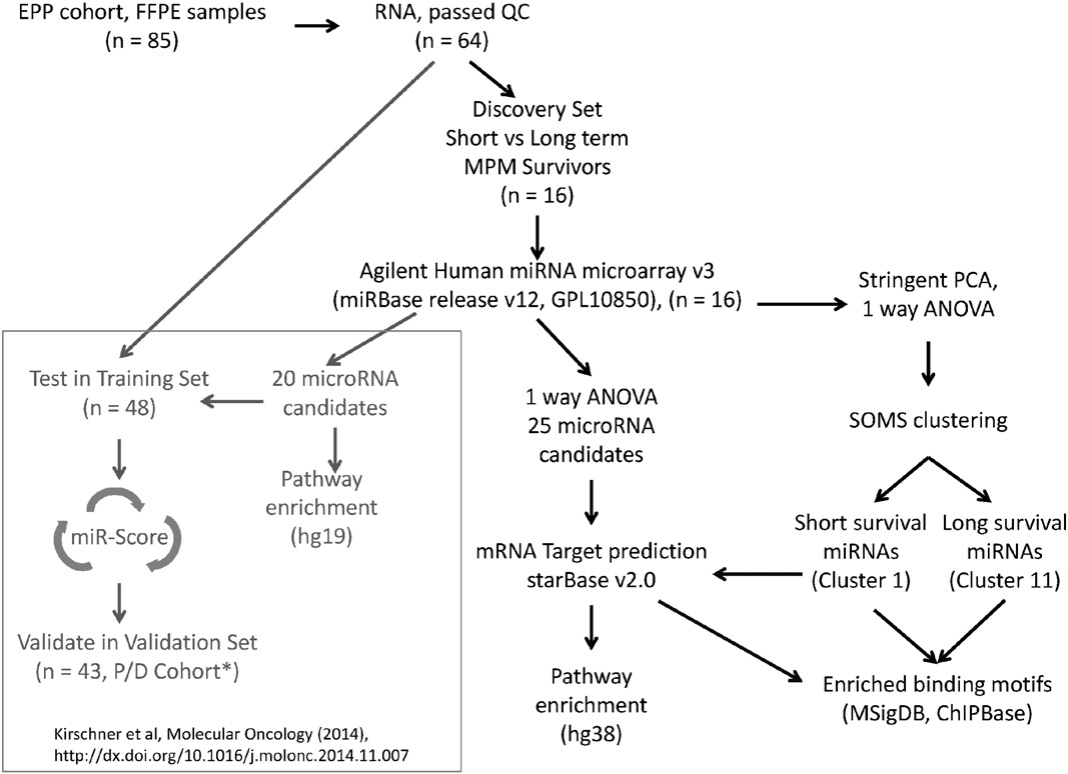
Analysis pipelines for miR-Score (Kirschner et al. [3]) and additional bioinformatics analysis. *This P/D cohort consisted of 75 patients but only 43 passed the criteria for RNA quality and quantity to be put forward for microRNA validation experiment.

The experimental design and analysis pipeline are outlined in Fig. 1. Briefly, we used 16 FFPE (formalin-fixed paraffin embedded) tumor samples from patients who underwent EPP (extrapleural pneumonectomy) [3], [5] to interrogate the prognostic value of genome-wide microRNA gene expression. The RNA samples were divided into 2 groups; 1) long survival, median = 53.7 months, n = 8 and 2) short survival, median = 6.4 months, n = 8. Agilent Human 8x15k miRNA microarrays (GPL10850) were utilized for microRNA transcriptome profiling. Agilent feature extraction v10.5 was used to extract fluorescence intensity.

### 2.1 Data processing

Total Gene Signals from the miRNA arrays were quantile normalized and log transformed to base 2. Further filtering was applied to exclude ones with gene expression of < 1 (Partek Genome Suite, v6.6). In comparison, Kirschner et al., 2015 used signal value of 1 as a threshold and normalized by shifting to the 90th percentile without applying baseline transformation. The intention here is to show that regardless of normalization methods used for the initial array data processing (Fig. 1), candidate microRNAs identified still hold true their association with the phenotype and in particular, the affected regulatory pathways where predicted genes are targeted by these microRNAs.

One-way ANOVA was used to examine differential gene expression between long and short survival (n = 16 in total). This analysis also assumes that long and short survival-related gene expression patterns are normally distributed and that the variance is approximately equal between the groups. Configuring the ANOVA in Partek Genome Suite (v6.6) enabled adjustment for systematic technical errors, such as batch processing of the RNA samples as well as the shortcomings of RNA extraction from FFPE blocks.

### 2.2 Visualization of gene expression and regulatory pathways

**Fig. 2:**
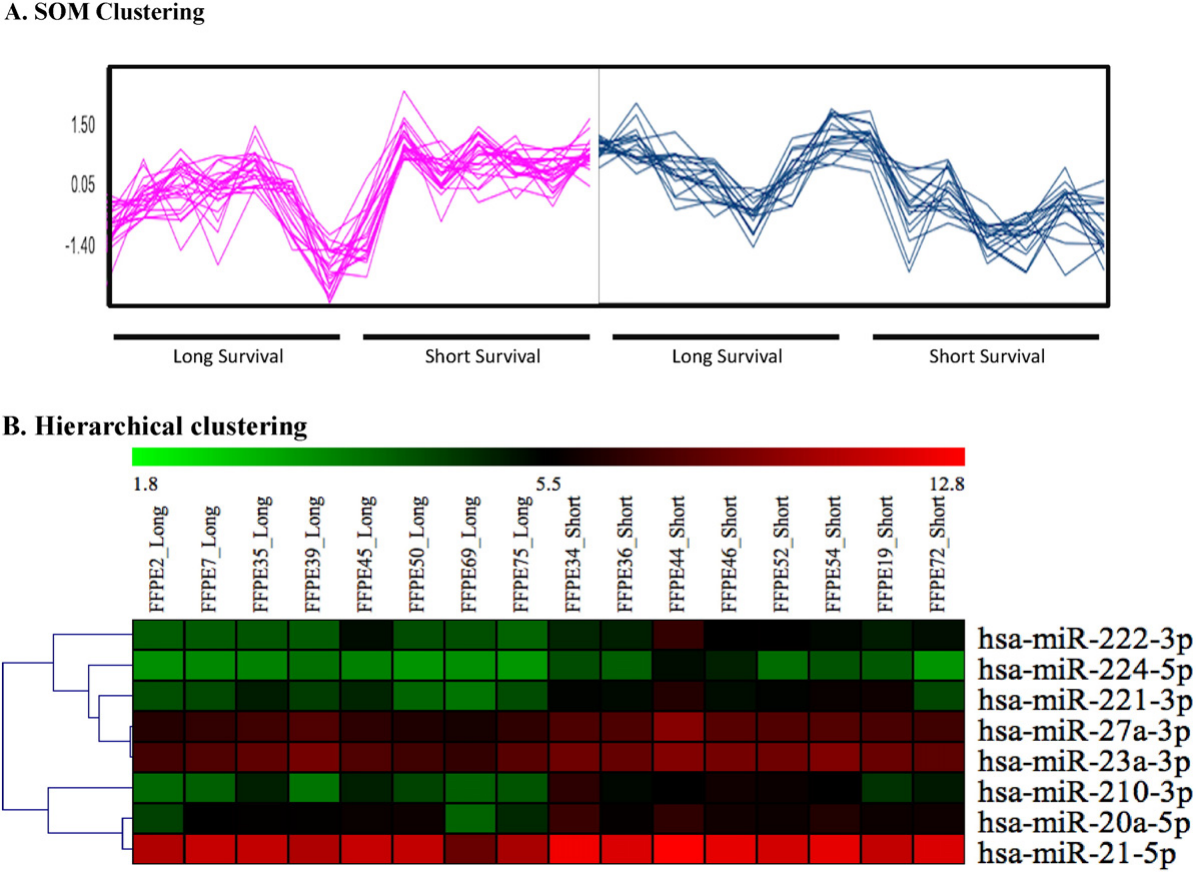
Visualization of microRNA microarray data. (A) SOM clustering of distinctive microRNA gene expression patterns in long vs short survival patients. (B) Hierarchical clustering showed distinctive gene expression pattern of candidate microRNAs to reflect coordinated regulation in long vs short survival patients.

To visualize the dataset, further analysis was carried out to identify distinctive gene expression patterns using Self-Organizing Map (SOM) clustering (map height = 4, map width = 3) (Fig. 2A). Cluster structure of this dataset in the form of non-linear mapping to a two-dimensional grid (SOM clustering) enabled us to explore the relationship and ultimately the function of these microRNAs. Hierarchical clustering (Fig. 2B) was also utilized (subdivide gene expression in similar vs different clusters) to identify significantly enriched functional categories. This is useful for unknown genes in the same cluster to enable us to infer a functional role. Two distinctive SOM clusters were identified and assigned to “Long Survival” and “Short Survival” based on the following criteria; 1) P < 0.05 differentially expressed, 2) Fold change > 1.5 and 3) correlation with enriched pathway based on predicted targets. We focused on 2 clusters, cluster 1 and cluster 11, as they showed distinct expression differences between long and short survival (Fig. 2A, Table 1).

Predicted target genes of microRNAs in the “Long Survival” and “Short Survival” clusters were extracted from miRDB [6] (Version 6.0, release date: August 2014) and starBase Version 2.0 [7]. Within starBase, predicted targets were extracted based on intersections with TargetScan [8], picTar [9], RNA22 [10], PITA [11] and miRanda/mirSVR [12]. This resolves some of the issues surrounding use of predicted targets from bioinformatics approaches where validation of targets leads to an estimated false positive rate of at least 20–40% [13], [14] and false negative rates of up to 50% [15], [16]. Although protocols such as HITS-CLIP [17] or PAR-CLIP [18] are considered in starBase for determining RISC occupancy on microRNAs, similar pitfalls still exist and we proceed with caution. The parameters used for starBase are medium stringency (≥ 2CLIP-Seq experiments supported the predicted miRNA target site).

To interrogate the data further, gene ontology (GO) enrichment analysis based on these target gene lists (Fisher's Exact test) was carried out to detect over-represented genes and to reflect enriched biological themes (biological process, molecular function and cellular component) as described [3]. Pathway enrichment analysis was carried out on this predicted target gene list to explore association of enriched regulatory pathways with patient survival outcome. This is based on Fisher's Exact test (P < 0.05), using the KEGG (Kyoto Encyclopedia of Genes and Genomes) [19] *Homo sapiens* hg19 Build reference genome as the background [3] as well as hg38 Build (Fig. 1). The aim was to characterize and compare the biological pathways best represented in both series. The higher the enrichment score the more enriched the genes are within a specific pathway (Table 2).

**Fig. 3:**
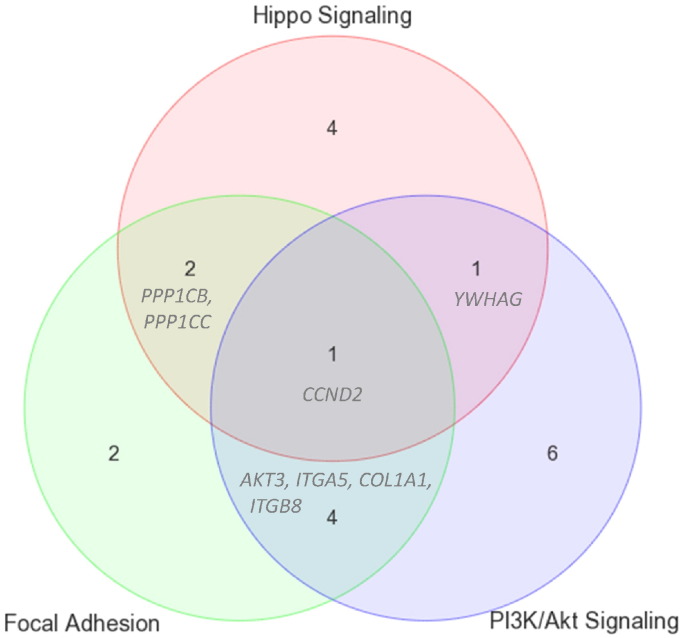
Venn diagram showing common gene targets within hippo signaling, PI3K/Akt signaling and focal adhesion pathways. The number denotes number of gene targets (extracted from starBase v2.0) in common with a specific pathway. For example, *CCND2*, target of hsa-miR-17-5p appears to be a common target to these three enriched pathways. At a systems level, design of microRNA-based therapeutic agents can then be deduced to either rescuing defective genes within a beneficial pathway and/or shutting down pathological gene(s) upstream of a regulatory cascade. For example; 1) *YWHAG* (hsa-miR-222-3p) and *CCND2* (hsa-miR-17-5p) are two genes that can be targeted to modulate gene expression in both PI3K/Akt signaling and hippo signaling pathways; 2) *CCND2*, *AKT3*, *ITGA5*, *COL1A1* and *ITGB8* can be targeted to affect PI3K/Akt signaling and focal adhesion pathways and 3) *CCND2*, *PPP1CB* and *PPP1CC* can be targeted by hsa-miR-17-5p, hsa-miR-23a-3p and hsa-miR-27a-3p respectively to modulate hippo signaling and focal adhesion pathways.

As shown from alternate data processing (Fig. 1) and discovery processes (Fig. 2), the results from these additional analyses support the robustness of miR-Score outlined in Kirschner et al. [3]. In the first instance, the directionality of fold change of the candidate microRNAs agrees (Table 1) where gene expression of hsa-miR-21-5p, hsa-miR-221-3p and members of the hsa-miR-17-92 cluster (hsa-miR-17-5p, hsa-miR-20a-5p) were higher in short survivors [3]. Furthermore, these microRNAs have been shown by others to be involved in PTEN regulation and modulate the PI3K/Akt signaling pathway [20], [21]. For visualization purposes, we compared genes targeted by these candidate microRNAs from pathways that have been found to be associated with cancer progression, tumor architecture and drug resistance [22], [23], i.e., PI3K/Akt signaling, hippo signaling and focal adhesion pathways (Fig. 3, Table 3). All of which have been implicated in mesothelioma biology [24].

### 2.3 Utilizing microRNA-based regulation to exert a systems effect

Of note, visualizing expression of microRNA and its targets within a pathway and identifying commonalities between these pathways (Fig. 3) enable further exploration, especially in terms of designing microRNA therapeutic targets [25]. Complex disease conditions such as heart failure and cancer are increasingly being recognized as the result of multiple dysfunctional pathways. Hence it is essential to change our approach towards disease treatment and develop new therapeutic strategies that seek to correct the overall network of pathways that has been distorted. At a systems level, the aim of either rescuing defective genes within a beneficial pathway and/or shutting down pathological gene(s) upstream of a regulatory cascade becomes clearer using this type of visualization approach [26], [27], [28]. For example, *CCND2*, a target of hsa-miR-17-5p, appears to be common to the three enriched pathways mentioned above. Inhibiting *CCND2* expression via hsa-miR-17-5p would thus modulate all three pathways and would have the potential to reduce tumor growth. According to starBase [7], *CCND2* is also a target of hsa-miR-15/16 and thus can potentially be targeted by TargomiRs, a novel microRNA-based treatment approach for MPM using microRNA-loaded EGFR antibody-targeted minicells (EDV™nanocells) [29]. In KEGG [19], *CCND2* is shown to be deeply entrenched in cell cycle progression, anti-apoptosis and pro-proliferation processes. Furthermore, in the case where gene expression of hsa-miR-21-5p, hsa-miR-221-3p, hsa-miR-17-5p and hsa-miR-20a-5p is higher in short survivors, an anti-microRNA approach [27] would be appropriate to restore the suppressed beneficial pathways.

In contrast, where down-regulated microRNAs are associated with a disease condition, a re-introduction of these microRNAs can be utilized to exert a systems effect in order to ameliorate dysregulated pathways. For example, in MPM, miR-16, miR-15a and miR-15b mimics were observed to have tumor suppressing properties [30] and subsequently a mimic based on the consensus sequence of the miR-15 family has shown signs of activity in a phase 1 clinical trial in MPM patients [29]. The assumption here is that detrimental pathways were rescued genome-wide. In comparison, a common seed-like sequence designed to exert microRNA-like regulation at 3′UTR region of unrelated genes [31], [32] can also have a systems effect.

Using the Molecular Signatures Database (MSigDB, now v5.1 [33]) to apply Gene Set Enrichment Analysis (GSEA) to differentially expressed genes from four MPM gene expression datasets, enabled us to identify enriched microRNA binding motifs that can be translated into potential druggable targets for MPM [34]. Here, GSEA on the twenty genes (Table 3) extracted from the enriched pathways revealed promoter regions (− 2 kb, 2 kb) around transcription start sites containing enriched binding motifs for transcriptional factors; MAZ (GGGAGGRR), PAX4 (GGGTGGRR), AP1 (TGANTCA) and FOXO4 (TTGTTT). All of which are implicated in MPM biology [35], [36], [37]. Using ChIPBase database (curated ChIP-seq (Chromatin immunoprecipitation [ChIP] with massive parallel DNA sequencing data) [38], we characterized further how these transcriptional factors interact with candidate microRNAs in the MPM system. Here, we found that Jun./AP1 binds to the promoter region (defined by ChIPBase as 5 kb upstream and 1 kb downstream) of the following microRNAs: hsa-miR-30c-1, hsa-miR-30e, hsa-miR-20a, hsa-miR-19a, hsa-miR-19b-1, hsa-miR-17, hsa-miR-18a, hsa-miR-92a-1, hsa-miR-93, hsa-miR-106b, hsa-miR-25, hsa-miR-210 and hsa-miR-95. This analysis implicated that targets of the microRNAs identified in our miR-Score study have the capacity to regulate other microRNAs (see Ooi et al. [28] for in-depth discussion on microRNA-transcriptional factor-microRNA interactions in a cardiovascular disease model [28]). Thus, the approach to identify enriched binding motifs and biological themes has merit in prioritizing genes for downstream validation, especially towards developing therapeutic agents. The complexity of the microRNA-transcriptional factor interactions is inferred here and we continue to utilize these approaches in our investigation into mesothelioma pathology.

## Figures and Tables

**Table 1:** Long survival microRNAs vs short survival microRNAs by SOM clustering analysis.

Cluster 1: short survival microRNA	p-Value (short vs long)	Fold-change (short vs long)	Fold-change (short vs long) (description)	Candidates microRNAs from [3]
hsa-miR-210-3p	0.0010555	4.02696	Short up vs long	x
hsa-miR-93-5p	0.00111034	3.3278	Short up vs long	x
hsa-miR-221-3p	0.0029257	6.45867	Short up vs long	x
hsa-miR-22-3p	0.00472295	1.61837	Short up vs long	
hsa-miR-151-5p	0.00943964	2.21248	Short up vs long	
hsa-miR-20a-5p	0.0102997	2.39401	Short up vs long	x
hsa-miR-92a-3p	0.0141019	1.69546	Short up vs long	x
hsa-miR-30e-5p	0.0196756	3.32221	Short up vs long	x
hsa-miR-146b-5p	0.021048	2.52768	Short up vs long	
hsa-miR-17-5p	0.0219565	3.21235	Short up vs long	x
hsa-miR-20b-5p	0.0256082	6.73325	Short up vs long	
hsa-miR-27b-3p	0.0295604	1.71494	Short up vs long	
hsa-miR-30c-5p	0.0319141	2.51445	Short up vs long	
hsa-miR-374a-5p	0.0386754	9.82208	Short up vs long	
hsa-miR-95-3p	0.046397	11.7385	Short up vs long	

Cluster 11: long survival microRNA	p-Value(short vs long)	Fold-change (short vs long)	Fold-change (short vs long) (description)	

hsa-miR-671-5p	0.00122393	− 2.28734	Short down vs long	
hsa-miR-188-5p	0.00362187	− 1.97211	Short down vs long	
hsa-miR-1469	0.00424181	− 3.8781	Short down vs long	x
hsa-miR-654-5p	0.00460829	− 12.2823	Short down vs long	
hsa-miR-622	0.00508603	− 1.974	Short down vs long	
hsa-miR-662	0.00675084	− 7.14828	Short down vs long	x
hsa-miR-1471	0.00688436	− 3.04317	Short down vs long	
hsa-miR-1183	0.00831059	− 1.9464	Short down vs long	
hsa-miR-431-5p	0.0100513	− 5.19376	Short down vs long	
hsa-miR-370-3p	0.0111777	− 2.9025	Short down vs long	
hsa-miR-345-5p	0.0122815	− 6.13592	Short down vs long	
hsa-miR-483-5p	0.012858	− 1.80356	Short down vs long	
hsa-miR-877-3p	0.0233915	− 1.64644	Short down vs long	
hsa-miR-1225-5p	0.025947	− 1.56253	Short down vs long	
hsa-miR-30c-1-3p	0.0262453	− 4.46393	Short down vs long	

**Table 2:** Pathway enrichment analysis of predicted targets of short vs long candidate microRNAs (enriched P < 0.001, extracted from starBase: http://starbase.sysu.edu.cn).

Pathway name	Enrichment score	Enrichment p-Value	# genes in list, in pathway	Pathway ID
Vasopressin-regulated water reabsorption	7.88895	0.000374863	5	kegg_pathway_57
Hippo signaling pathway	6.25988	0.00191147	8	kegg_pathway_96
Pancreatic cancer	5.794	0.00304576	6	kegg_pathway_249
Vascular smooth muscle contraction	5.71464	0.00329734	7	kegg_pathway_118
Chronic myeloid leukemia	5.57127	0.00380564	6	kegg_pathway_69
Hepatitis B	5.50279	0.00407539	8	kegg_pathway_89
Focal adhesion	5.47739	0.00418024	9	kegg_pathway_188
Dilated cardiomyopathy	5.46448	0.00423454	6	kegg_pathway_263
Regulation of actin cytoskeleton	5.43195	0.00437454	9	kegg_pathway_139
PI3K-Akt signaling pathway	5.03748	0.00649008	12	kegg_pathway_262
Shigellosis	4.84404	0.00787516	5	kegg_pathway_82
HTLV-I infection	4.74205	0.00872078	10	kegg_pathway_190

**Table 3:** Gene targets of candidate microRNAs from PI3K/Akt signaling, hippo signaling and focal adhesion pathways (enriched P < 0.001, extracted from starBase: http://starbase.sysu.edu.cn).

PI3K-Akt signaling pathway			
Gene targets	RefSeq/Gene name	microRNA	Position
*COL1A1*	NM_000088//collagen, type I, alpha 1	hsa-let-7i-5p	chr17:48262068-48262074[−]
*ITGB8*	NM_002214//integrin, beta 8	hsa-miR-106b-5p	chr7:20449851-20449858[+]
*RBL2*	NM_005611//retinoblastoma-like 2	hsa-miR-106b-5p	chr16:53524846-53524853[+]
*CCND2*	NM_001759//cyclin D2	hsa-miR-17-5p	chr12:4410143-4410149[+]
*CDKN1A*	NM_000389//cyclin-dependent kinase inhibitor 1A (p21, Cip1)	hsa-miR-17-5p	chr6:36654725-36654731[+]
*JAK1*	NM_002227//Janus kinase 1	hsa-miR-17-5p	chr1:65298950-65298956[−]
*EFNA3*	NM_004952//ephrin-A3	hsa-miR-210-3p	chr1:155059817-155059823[+]
*YWHAG*	NM_012479//tyrosine 3-monooxygenase/tryptophan 5-monooxygenase activation protein gamma	hsa-miR-222-3p	chr7:75956151-75956157[−]
*ITGA5*	NM_002205//integrin, alpha 5 (fibronectin receptor, alpha polypeptide)	hsa-miR-25-3p	chr12:54789081-54789088[−]
*CSF1*	NM_000757//colony stimulating factor 1 (macrophage)	hsa-miR-27a-3p	chr1:110472264-110472271[+]
*KITLG*	NM_000899//KIT ligand	hsa-miR-27a-3p	chr12:88890807-88890814[−]
*AKT3*	NM_001206729//v-akt murine thymoma viral oncogene homolog 3	hsa-miR-93-5p	chr1:243667403-243667409[−]
Hippo signaling pathway			
*GDF6*	NM_001001557//growth differentiation factor 6	hsa-let-7i-5p	chr8:97154690-97154697[−]
*TGFBR1*	NM_001130916//transforming growth factor, beta receptor 1	hsa-let-7i-5p	chr9:101911662-101911669[+]
*CCND2*	NM_001759//cyclin D2	hsa-miR-17-5p	chr12:4410143-4410149[+]
*FRMD6*	NM_001042481//FERM domain containing 6	hsa-miR-20a-5p	chr14:52194908-52194915[+]
*TGFBR2*	NM_001024847//transforming growth factor, beta receptor II (70/80 kDa)	hsa-miR-21-5p	chr3:30733297-30733303[+]
*YWHAG*	NM_012479//tyrosine 3-monooxygenase/tryptophan 5-monooxygenase activation protein gamma	hsa-miR-222-3p	chr7:75956151-75956157[−]
*PPP1CB*	NM_002709//protein phosphatase 1, catalytic subunit, beta isozyme	hsa-miR-23a-3p	chr2:29022976-29022982[+]
PPP1CC	NM_002710//protein phosphatase 1, catalytic subunit, gamma isozyme	hsa-miR-27a-3p	chr12:111158440-111158447[−]
Focal adhesion			
*COL1A1*	NM_000088//collagen, type I, alpha 1	hsa-let-7i-5p	chr17:48262068-48262074[−]
*ITGB8*	NM_002214//integrin, beta 8	hsa-miR-106b-5p	chr7:20449851-20449858[+]
*MAPK9*	NM_002752//mitogen-activated protein kinase 9	hsa-miR-106b-5p	chr5:179663017-179663023[−]
*CCND2*	NM_001759//cyclin D2	hsa-miR-17-5p	chr12:4410143-4410149[+]
*PPP1CB*	NM_002709//protein phosphatase 1, catalytic subunit, beta isozyme	hsa-miR-23a-3p	chr2:29022976-29022982[+]
*ITGA5*	NM_002205//integrin, alpha 5 (fibronectin receptor, alpha polypeptide)	hsa-miR-25-3p	chr12:54789081-54789088[−]
*PPP1CC*	NM_002710//protein phosphatase 1, catalytic subunit, gamma isozyme	hsa-miR-27a-3p	chr12:111158440-111158447[−]
*VCL*	NM_003373//vinculin	hsa-miR-34a-5p	chr10:75878809-75878815[+]
*AKT3*	NM_001206729//v-akt murine thymoma viral oncogene homolog 3	hsa-miR-93-5p	chr1:243667403-243667409[−]
